# The Critical Periphery in the Growth of Social Protests

**DOI:** 10.1371/journal.pone.0143611

**Published:** 2015-11-30

**Authors:** Pablo Barberá, Ning Wang, Richard Bonneau, John T. Jost, Jonathan Nagler, Joshua Tucker, Sandra González-Bailón

**Affiliations:** 1 Center for Data Science, New York University, New York, New York, 10003, United States of America; 2 Mathematical Institute and Oxford Internet Institute, University of Oxford, Oxford, OX26GG, United Kingdom; 3 Center for Genomics and System Biology, New York University, New York, New York, 10003, United States of America; 4 Simons Center for Data Analysis, Simons Foundation, New York, New York, 10010, United States of America; 5 Department of Psychology, New York University, New York, New York, 10003, United States of America; 6 Department of Politics, New York University, New York, New York, 10012, United States of America; 7 Annenberg School for Communication, University of Pennsylvania, Philadelphia, Pennsylvania, 19104, United States of America; Arizona State University, UNITED STATES

## Abstract

Social media have provided instrumental means of communication in many recent political protests. The efficiency of online networks in disseminating timely information has been praised by many commentators; at the same time, users are often derided as “slacktivists” because of the shallow commitment involved in clicking a forwarding button. Here we consider the role of these peripheral online participants, the immense majority of users who surround the small epicenter of protests, representing layers of diminishing online activity around the committed minority. We analyze three datasets tracking protest communication in different languages and political contexts through the social media platform Twitter and employ a network decomposition technique to examine their hierarchical structure. We provide consistent evidence that peripheral participants are critical in increasing the reach of protest messages and generating online content at levels that are comparable to core participants. Although committed minorities may constitute the heart of protest movements, our results suggest that their success in maximizing the number of online citizens exposed to protest messages depends, at least in part, on activating the critical periphery. Peripheral users are less active on a *per capita* basis, but their power lies in their numbers: their aggregate contribution to the spread of protest messages is comparable in magnitude to that of core participants. An analysis of two other datasets unrelated to mass protests strengthens our interpretation that core-periphery dynamics are characteristically important in the context of collective action events. Theoretical models of diffusion in social networks would benefit from increased attention to the role of peripheral nodes in the propagation of information and behavior.

## Introduction

Do social media help raise awareness of political causes or do they simply encourage “feel-good” politics with hollow consequences? The wave of international protests that began in 2011 spurred controversy about whether online networks serve or impede the goals of mobilization [1–3]. The question is revived every time mass mobilizations ripple through social media, most recently during the umbrella revolution staged by protesters in Hong Kong in 2014–2015 [4]. The debate often arises when the daring minority of highly committed protesters is compared with the less heroic majority of followers who may risk relatively little, posting messages comfortably from a distance. This depiction, however, fails to acknowledge the complex forces that are at play in the current media environment, including the synergies that both core and peripheral participants create in the process of starting and scaling up visibility of the protest movement. In this article we characterize these synergies and devise a strategy to measure the relevance of the immense periphery, gauging their role in contributing an important mobilization resource–the ability to disseminate information about the protest events.

Research on collective action has long emphasized the importance of resource mobilization to understand the success of social movements [5]. Generalized discontent is a precondition for social protests to arise, but the ability to reach, recruit, and organize participants is what allows initial sparks to spread like ‘wildfires’, to use one common analogy in historical accounts of contentious politics [6]. Resource mobilization theories often measure success by the number of participants mobilized, which is assumed to increase as a function of the number of participants who are already active [7, 8]. Critical mass theories aim to identify the factors that cause participation to become self-sustaining [9, 10]; that is, they focus on how and why the number of participants reaches a tipping point—the moment when the spark becomes a wildfire.

Social networks are central to the analytical treatment of these questions: they provide the structure of interdependence that shapes individual decision-making (i.e., whether to join a given protest), and they channel the propagation of activation signals (i.e., how many other people are already participating). In other words, networks hold the key to decoding the social logic of protests. Most of the insights gained so far on how networks mediate collective action derive from simulations and mathematical models that consider a short list of network properties; in particular, prior research has emphasized the effects of density, degree distribution, degree centralization, network size, and the prevalence of weak ties [10–14]. In addition, these formal models often assume stylized networks that are convenient analytically but not very useful for accurately depicting real-world social networks, which are far too complex to be fully characterized in terms of the properties just listed. For instance, the core-periphery structure seen in many empirically observed networks [15] has been neglected in work aimed at testing critical mass theories. Fortunately, the increasing availability of data logs tracking large-scale communication provides new research opportunities to analyze dynamics that are obscured by more traditional data sources [16–18]; in particular, communication dynamics in social media help to reveal the contribution that peripheral actors make to collective action efforts.

## Data and Methods

To address these questions, we make use of three datasets tracking Twitter communication concerning three protest events spanning different languages and political contexts. The first dataset tracks communication about Twitter activity around the protests that emerged in Istanbul’s Taksim Gezi Park in May 2013. The protests started as a sit-in against urban development plans but soon escalated into massive antigovernment demonstrations. We sampled activity for the period May 31 to June 29, 2013 using Twitter’s streaming API, querying for messages that contained hashtags related to the protests. We selected these terms by observing the trending topics in Turkey and in consultation with local experts (see [19] for the full list of terms). Our selection of keywords is consistent with other studies of the Turkish protests [20]. We collected a total of 30,019,710 messages sent by 2,908,926 unique users. Although this dataset is likely to exclude many tweets related to the protest that did not mention one of the main protest hashtags, we believe this is decision should not affect our results (see S1 Text for a discussion of potential biases related to our data collection process). We obtained the number of followers associated with each sampled user. We also have information about the location of some users, which we obtained from tweets that contained the geographic coordinates from which they were sent. This information will allow us to identify users who sent at least one tweet in the area around Taksim Gezi Park in Istanbul, which we consider an indication that they participated in the protest. Finally, we parsed the messages to identify retweets, which allowed us to reconstruct the network of information flow around the protest.

The second and third datasets track Twitter activity around the international call for action “United for Global Change,” a demonstration planned for May 12, 2012. This was the second call for action under the same slogan, the first having been organized in October 15, 2011 as part of the wave of protests that started with the Arab Spring, and continued with the Spanish Indignados and the Occupy movement. This second call for action was less successful at mobilizing people and raising public awareness. We sampled activity for the period April 30 to May 30, 2012 using the search API, querying for messages that contained hashtags related to the Indignados movement (identified in previous work [21, 22]) and variations of the word Occupy*. A total of 606,625 messages sent by 125,219 unique users were collected. As with the Turkish dataset, we also obtained the number of followers associated with each sampled user and parsed the messages to identify retweets, which allowed us to reconstruct the networks we will examine in our analysis.

To determine whether the core-periphery dynamics we identify are characteristic of protest communication or they are, instead, simply widespread in communication around all types of conversations on Twitter, including those unrelated to protests, we replicated our analyses with two additional datasets. The first contained messages related to the 2014 Academy Awards. We sampled activity for the period March 2, 2014 at 2pm ET to Wednesday March 5 at 2pm ET using the streaming API, querying for the keywords “oscars”, “#oscars2014”, and “academy awards”. We collected a total of 7,527,157 tweets sent by 3,910,627 unique users. The second dataset is formed by messages related to the debate about raising the minimum wage in the United States. We sampled activity for the period February 3, 2014 to February 2rd, 2015 using the streaming API, querying for the keywords “minimum wage”, “minimum hourly wage”, “raise the wage”, “#giveamericaaraise” (and other less frequent hashtags). In total, we collected 2,957,847 tweets sent by 1,199,414 unique users.

We analyzed the network of retweets among participants, which we defined as users who employed relevant hashtags. We treat retweets as the directed ties that link users in a network of information exchange, where each edge originates in the user who retweets and ends in the user whose message is retweeted. Table 1 shows high-level statistics for the five networks analyzed. The indegree distributions, reciprocity levels, and degree correlation all suggest a hierarchical, core-periphery structure with a minority of participants at the core. For example, the maximum indegree (number of times retweeted) is 181,387, whereas the maximum outdegree (number of retweets sent) is 2,397.

**Table 1:** Summary statistics for the three retweet networks analyzed (largest weakly connected component).

	Gezi	Occupy	Indignados	Oscars	Minimum Wage
Nodes	1,935,911	30,708	49,534	2,800,880	721,660
Arcs	15,761,311	80,967	124,519	3,925,396	1,310,384
Average degree	16	5	5	3	4
Average indegree	8	3	3	1	2
Max indegree	181,387	2,092	3,898	918,968	96,669
Average outdegree	8	3	3	1	2
Max outdegree	2,397	902	264	514	942
Reciprocity	0.011	0.041	0.031	0.002	0.082
Clustering	0.091	0.147	0.125	0.066	0.094
Degree correlation	-0.063	-0.081	-0.108	-0.110	-0.101

In addition to the network of RTs, we use the total number of messages containing any of the relevant hashtags for each collection (regardless of whether they are RTs) as node attribute information. We also count, for each node, the total number of followers. These two attributes are the basis for our measurement of activity and reach.

**Fig 1:**
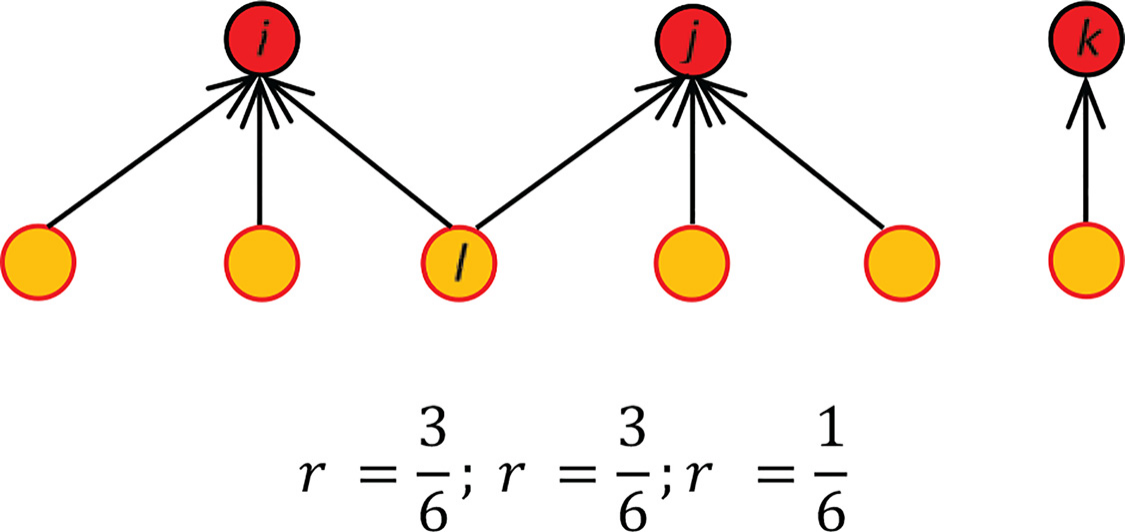
Reach measured as a fraction of all followers. In this schematic representation, there are three protest participants that accumulate six unique followers. The relative reach of each participiant (nodes in red) is the fraction of their direct followers over the total available in the system (nodes in orange). We normalize these counts to fall in the interval [0,1] for the three networks.

We measure *reach* as the fraction of followers that every participant has over the total number of followers in the network. As Fig 1 illustrates, this means that some followers (like node *l*) are counted more than once. Because of the clustering in the Twitter network [23], our count probably overestimates the number of unique users who are exposed to protest information; however, prior research on complex contagion and the psychological effects of repeated exposure suggests that the adoption of a given behavior (such participation in a protest) is more likely after individuals become familiar with the stimuli [13, 14, 24]. Psychological research also shows that familiarity with statements increases the likelihood that those statements will be judged to be valid and true [25, 26]. By counting the same followers more than once, we tap into the mobilization potential of repeated exposure–all else equal, node *l* will be more likely to join the flow of protest communication than other followers who are only exposed to protest information from a single source. For the same reason, we assume that a similar feedback mechanism exists among participants who are following other participants: the more information they are exposed to, the more engaged they are likely to become. Although we cannot disentangle any causal relationships given the observational nature of our data, this positive feedback is likely to drive (at least in part) the higher density of ties we identify at the core of the network, where participants are on average more active in posting messages and retweets.

Overall, our measure of reach is roughly comparable to more standard measures of audience share in media market studies (e.g., Nielsen ratings). We measure how many Twitter users are exposed to protest-related information through at least one of the users they decided to follow. This is similar to measuring the share of households with their televisions or radios on that are tuned to a particular channel. We cannot be certain that followers are reading the protest messages that appear in their feeds, much as rating measurements provide no guarantee that members of a household tuning into a particular program are actually paying attention.

**Fig 2:**
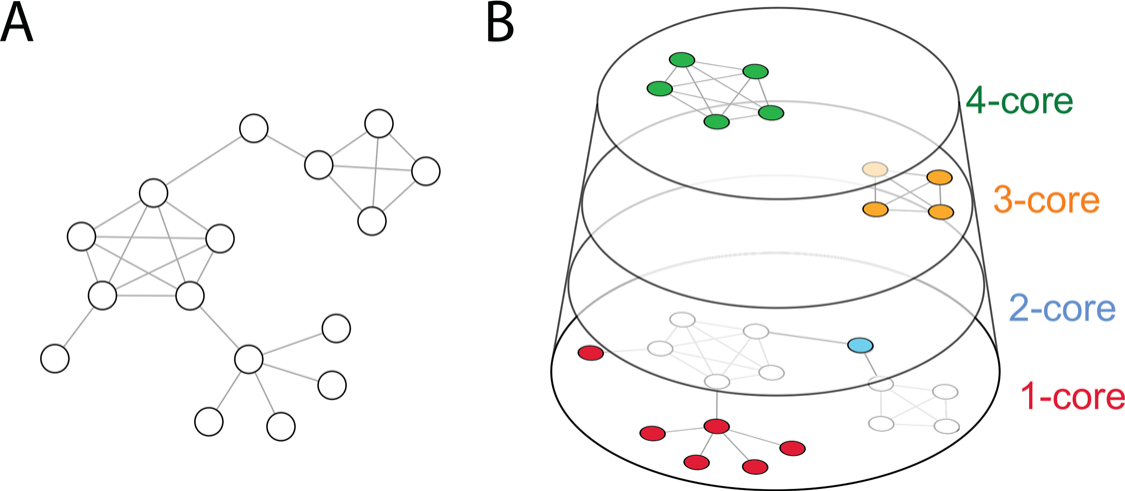
Schematic representation of the *k*-core decomposition for a random network with N = 16 vertices and E = 24 edges. This technique recursively prunes the network to remove nodes with the lowest degree. The coreness of a vertex is *k* if it belongs to the *k*-core but not to the (*k*+1)-core.

We identify core and peripheral participants using the *k*-core decomposition technique, which partitions a network in nested shells of connectivity [27, 28]. The *k*-core of a graph is the maximal subgraph in which every vertex has at least degree *k*. In our case, degree relates to the number of retweets made or received. The *k*-core decomposition is a recursive approach that progressively trims the least connected nodes in a network (i.e. those with lower degree) in order to identify the most central ones. Fig 2 illustrates the *k*-core decomposition of a random graph with 19 vertices and 24 edges. Node degree is in the range of 1 to 5, but there are only four cores. Since the method is recursive, some of the nodes with degree 5 end up being classified in lower *k*-shells. Nodes classified in higher *k*-shells not only have higher degree: they are also connected to nodes that are central as well. Low engagement participants are classified in lower *k*-shells, and they form the periphery of the network.

We use the network of retweets to distinguish between core and peripheral participants. However, not all messages relevant in the dissemination of protest-related information are retweets to other users. This is clearly the case for individuals at the protests themselves, who are perfectly capable of producing original tweets with relevant information; this even holds for those not at the protests, who may write original tweets summarizing what they have learned from friends, colleagues, and other sources of new and traditional media. Consider someone watching a Twitter feed and seeing five tweets in a row related to the use of teargas. This person could choose to retweet one of those five tweets, but could just as easily tweet something along the lines of “multiple tweets reporting teargas at #gezipark”. We therefore measure *protest activity* as the total number of messages that contained at least one protest-related hashtag, regardless of whether that message was a retweet. For the non-protest-related network, we also measure activity as the total number of messages containing at least one hashtag or keyword related to the event. We produce three metrics: one at the level of individual participants, one at the level of *k*-cores, and one at the level of the entire network, which we use to assess the share that each participant and *k*-core contributes to overall activity volume.

Our analyses assess the impact of peripheral users by simulating how removing them from the network would affect the two outcome variables, audience and reach, in comparison to a random benchmark. This benchmark is based on a random assignment of *k*-core values (sampled without replacement, averaged over 10,000 permutations). The benchmark can, in fact, be interpreted as a line of perfect equality, that is, it plots what would happen if all *k*-cores contributed the same amount to overall activity and reach. The farther the reach and activity curves fall from this line, the more unequally distributed these resources are in the network.

## Results

**Fig 3:**
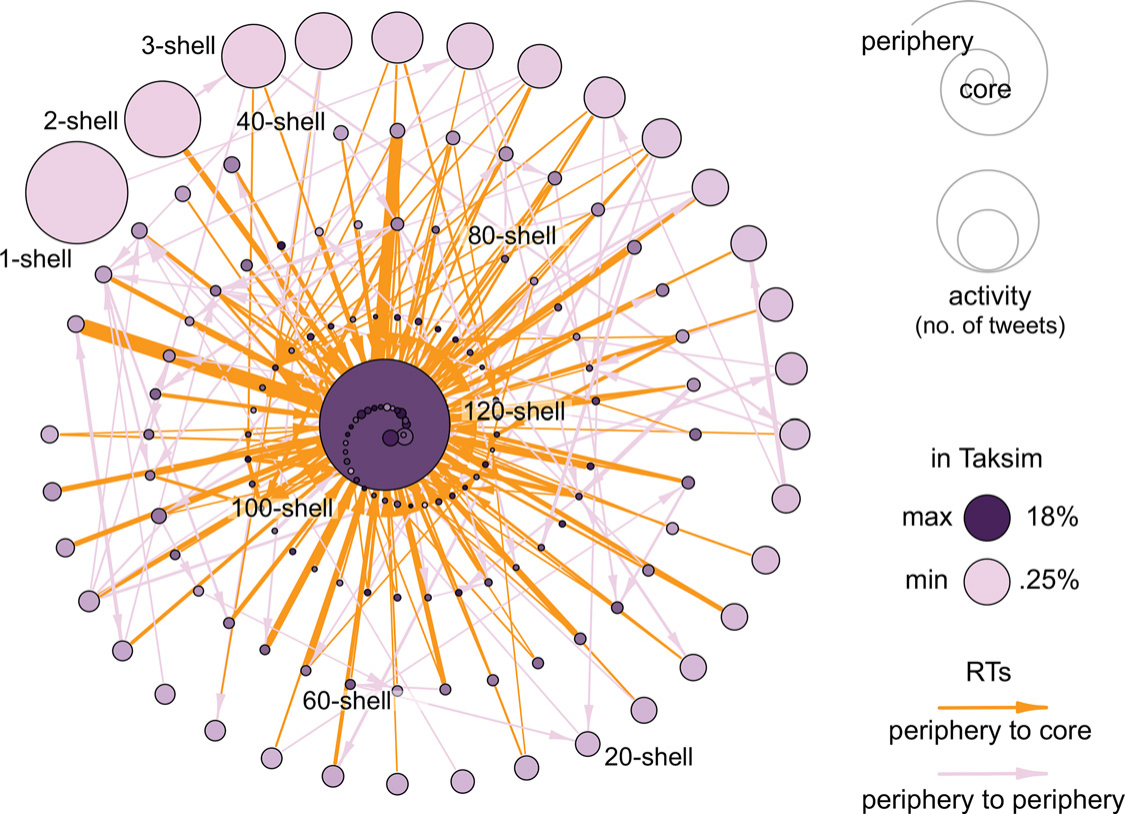
*K*-core decomposition of the network of retweets that emerged during the 2013 Taksim Gezi Park protests in Turkey (see S1 Text). Participants have been grouped in their corresponding *k*-shells, here represented by nodes. Lower *k-*shells contain participants at the periphery of the network; higher *k*-shells contain core participants. Node size is proportional to aggregated activity, measured as total number of protest messages (not just retweets). Arcs indicate retweeting activity, and their width is proportional to normalized strength (arcs with lower strength have been filtered to improve the visualization of the network). The darkness of nodes is proportional to the percentage of participants who reported being in the Taksim Gezi Park (the geographical epicenter of the protests), as indicated by the geographic information attached to their tweets. Most of these participants are at the core of the network where most RTs are also sourced from, thus allowing information to flow from the core to the periphery.

Fig 3 illustrates the *k*-core decomposition of the communication network that emerged during the Turkish protests. This network offers a simplified map of participants’ interactions and information exchange. The group with the highest percentage of users who reported being at the Taksim Gezi Park (the geographical epicenter of the protests) constitutes the core of the network, where most of the RTs are also directed—or sourced—from. This indicates that information flowed largely from the core to the periphery, allowing many participants who were not on the streets to be informed in real time of activity on the ground. Access to timely information through online networks is especially important in the Turkish context, where mainstream media is heavily controlled and, in the early stages of the protests, was used to divert attention away from the events happening in Gezi Park. Famously, the major news network broadcasted a penguin documentary rather than covering the massive protest mobilization and confrontation with police taking place in the park [29].

**Fig 4:**
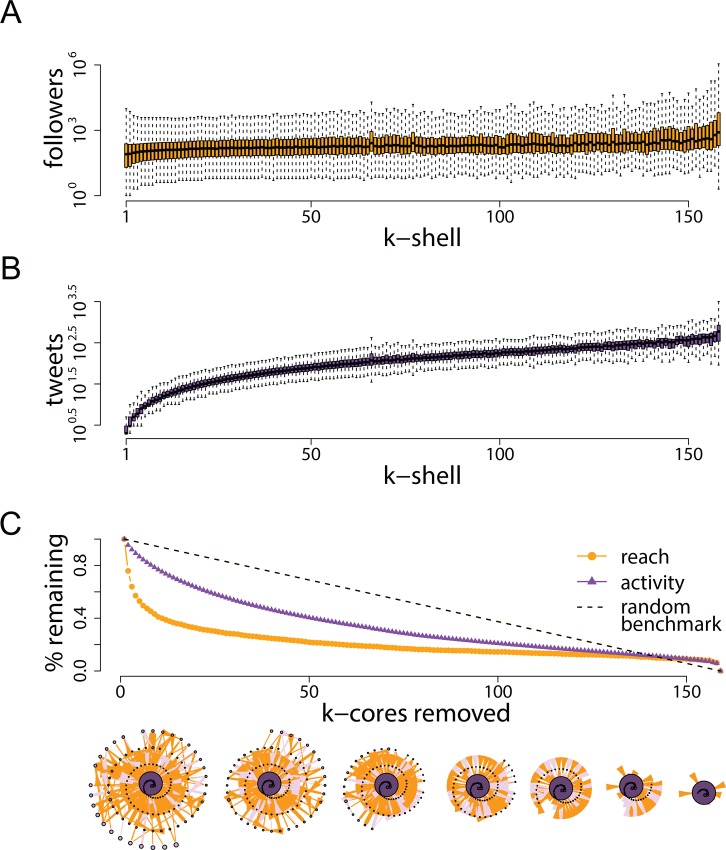
Audience size and activity levels across *k*-cores. Panel A shows the distribution in number of followers (or reach) across *k*-shells. Panel B plots the distributions in number of tweets sent (or activity) across k-shells. Panel C shows the effects on overall reach and activity of removing *k*-cores progressively, starting from the lowest or most peripheral as illustrated by the networks below the horizontal axis. Removing the first five *k*-cores results in a drop of slightly more than 50% in total reach capacity, suggesting that the sphere of influence of core participants is much reduced without peripheral contributors. The random benchmark is based on 10,000 permutations of the data where assignment to *k*-cores is randomly re-shuffled; this benchmark can be interpreted as a line of perfect equality, i.e. a scenario in which all *k*-cores contribute the same amount to overall activity and reach.

Fig 4 shows that, on average, participants across all *k*-shells have a similar number of followers (panel A). Peripheral users are substantially less active on a *per capita* basis than core participants (panel B), but their power lies in their numbers: there are so many more of them (38% in the 1-shell or 15% in the 2-shell vs .13% in the 158-shell, see S1 Text) that their aggregate contribution to the exchange of information is, in fact, greater than that of core participants. Panel C in Fig 2 shows how activity levels and overall reach would vary if outer *k*-cores were progressively removed. As described in the previous section, we define *reach* as the aggregate size of participants’ audience, and *activity* as the total number of protest messages published, whether or not they are retweets. Removing the lowest five *k*-cores results in a drop of slightly more than 50% in reach; overall activity levels drop less prominently, but those messages now have a significantly lower chance of being seen or retweeted. Thus despite the relative lack of reach of any particular individual in these last five *k*-cores, the reach of the active core participants would have been *substantially* diminished by the absence of these peripheral members of the network.

**Fig 5:**
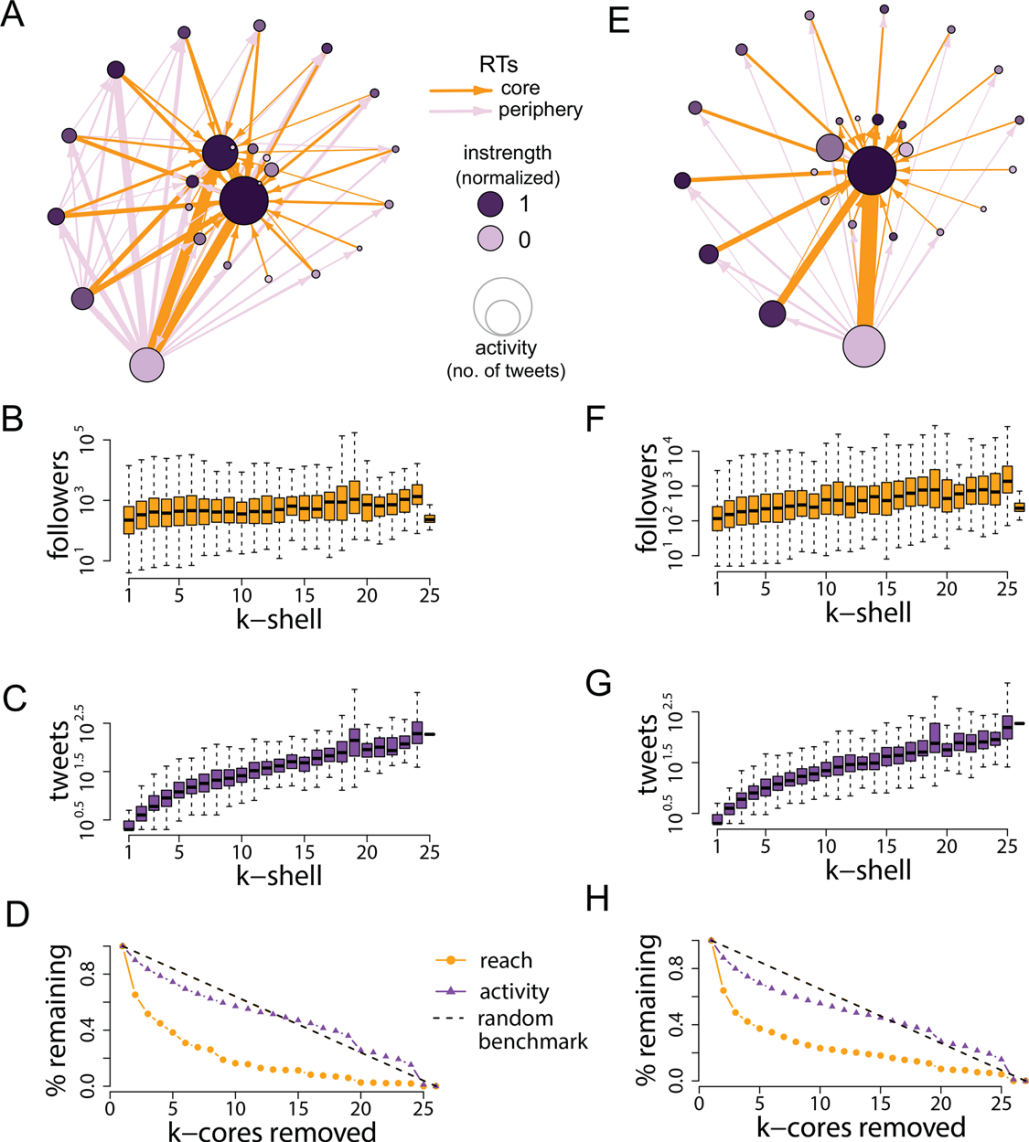
Core-periphery analysis for the Occupy and Indignados networks. Panels A and E visualize the connections across *k*-cores (arcs with lower strength have been filtered to improve visualization). Unlike the Turkish case, these protests did not have a clear epicenter, so nodes are colored in proportion to the number of retweets received (i.e. normalized instrength). Core-periphery dynamics are, however, similar to the Turkish case: most of the information flows from the core to the periphery, where users are significantly less active on a per capita basis but who, on the aggregate, contribute a similar volume of messages. Panels D and H show that removing the three outer *k*-cores results in a drop of audience of about 50%. The random benchmark is, again, based on 10,000 permutations of the data and it can be interpreted as a line of perfect equality.

We replicated the same analyses with two more datasets related to protest events: one tracking communication related to the Occupy Wall Street movement, the other tracking the Spanish Indignados, both around the same global call for action in May of 2012. Panels A and E in Fig 5 illustrate the *k*-shells and their connectedness. Unlike the Turkish case, these protests did not have a clear epicenter, so nodes are colored in proportion to the number of retweets received or instrength [30], normalized to range between 0 and 1. The networks are smaller and sparser than in the Turkish case (see Table 1), resulting in fewer *k*-cores; however, the core-periphery dynamics are similar: most of the information flows from the core to the periphery, where users are significantly less active on a *per capita* basis but who contribute as many messages at the aggregate level. Panels D and H show that removing the three outer *k*-cores results, once again, in a drop in audience reach of approximately 50%.

**Fig 6:**
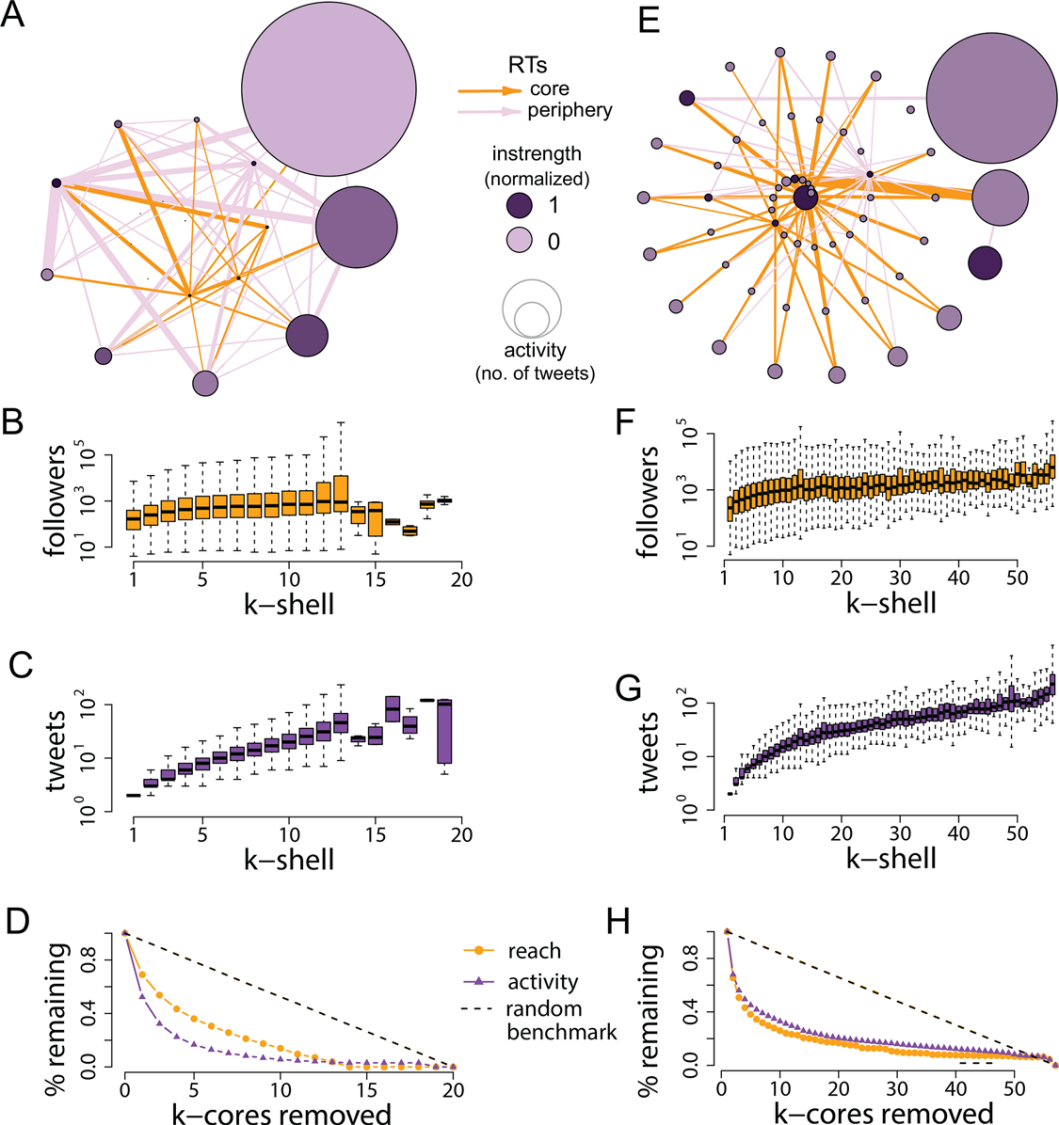
Audience size and activity levels across *k*-cores for the Oscars network (panels A-D) and the minimum wage network (panels E-H). Arcs with lower strength have been filtered to improve network visualization.

In contrast, Fig 6 reproduces the *k*-core analyses for the two networks that are not related to protest events. The activity that comes from the core is significantly lower compared to the activity that arises from the periphery, which is disproportionately larger not only in terms of number of users but also in terms of aggregated number of messages. This is especially the case for the Oscars network, where the core is virtually invisible under the shadow of the periphery. What this means is that, as panel D shows, removing the first few cores has a higher impact on activity than on reach–exactly the opposite of what happens in the protest networks. The core is slightly more prominent in the minimum wage network, but again, removing the first few cores has a similar impact on activity and reach (panel H).

**Fig 7:**
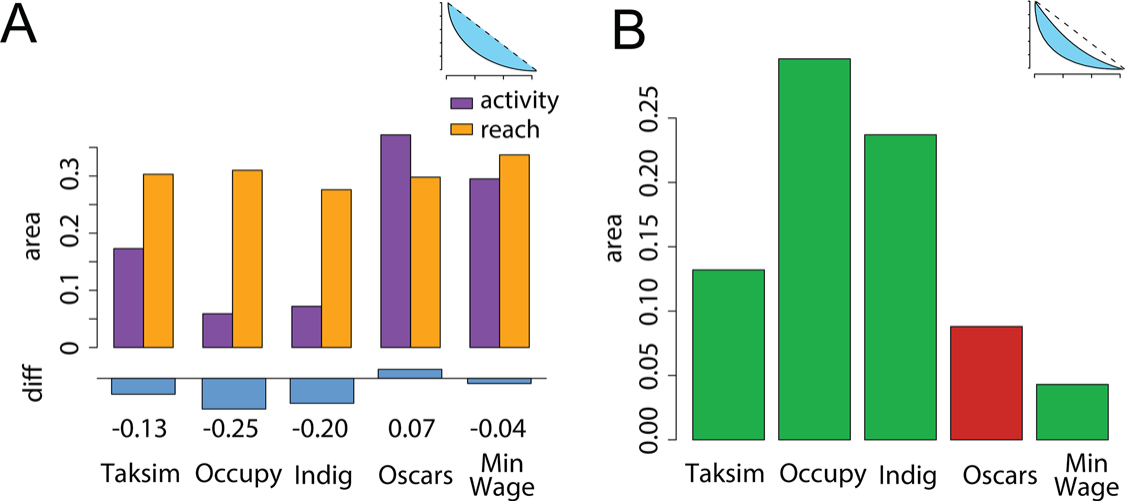
Area above reach and activity curves, bounded by random benchmark (panel A) and area between reach and activity curves, which serves as an index of core-periphery dynamics (panel B). The diagrams on the upper-right corner indicate the areas that were computed in each panel. The Oscars dataset is the only one in which removing the lower cores has a greater impact on activity than reach–hence its different color in the barplot of panel B.

These differences can be best measured as the area between curves, illustrated in Fig 7. Panel A shows the area between the activity and reach curves, and the random benchmark, which, again, can be interpreted as a line of equality where the contribution of all *k*-cores is perfectly equal. The areas are more similar for the two datasets that are not protest-related. Furthermore, the only dataset that does not track political communication is the only one in which the area above activity is larger, signifying that the network is more hierarchical in the distribution of content creation (with most of it concentrated in the periphery). Panel B plots the area between the activity and reach curves for the five datasets; this measure serves as an index of core-periphery activity. Again, the non-protest networks show a visible difference: the two curves are closer, signaling a lack of division of labor between core and peripheral users.

This comparison strengthens our interpretation that core-periphery dynamics are especially important in the context of political protests. The committed minorities at the core of collective action events generate a significant amount of information, and the peripheries help to expand the audiences for those messages. The communication networks we analyzed suggest that despite the low individual impact of peripheral participants, they offer–as a collective–potentially valuable resources to amplify the reach and increase the number of citizens who are able to learn about the actions of the core protestors.

## Discussion

Our findings suggest that peripheral users in online protest networks may be as important in expanding the reach of messages as the highly committed minority at the core. We do not find equivalent patterns in datasets that concern topics unrelated to political protests. Peripheral users possess potentially valuable mobilization resources that greatly increase the number of online individuals who are exposed to protest messages initiated by core participants. Given the power of the Turkish mainstream media to censor information, Gezi Park protest organizers needed to spread news through online networks, and they did so successfully: the protests became international news in a matter of hours [31]. Protestors at the core of this network–a large proportion of whom were actual protesters on the ground, as evidenced by the location of the messages they shared on Twitter (see S1 Text)–would have had a much harder time reaching such a large online audience without the critical periphery of low-activity users. Our analysis suggests that, because of their numbers, peripheral users managed to generate a great deal of activity in Twitter related to this cause.

This does not imply that social media can always be used to activate the critical periphery to the same degree. In some cases, the periphery might not be large enough to increase the audience of protest messages and, as a result, fail to raise sufficient awareness of the actions taken by the committed minority. Indeed, the global demonstrations planned for May of 2012 were not as successful in terms of their media impact as the first wave of Indignados and Occupy protests in 2011 [32]. Decades of research on resource mobilization shows that there are many factors involved in the success of collective action [33]. Social media is just one instrument that needs to be played effectively.

Independent of the social and material constraints that might restrict mobilization, our findings demonstrate that relatively low commitment participants–who are often derided as feel-good activists or “slacktivists” [2, 3]–are potentially very important as a collective. By expanding the audience of messages sent by the committed minority, the periphery can amplify the core voices and actions, and thus provide a way for larger numbers of online citizens to be exposed to news and information about the protest, even (or especially) in the absence of mass media coverage. The availability of information about protest events is important because it can increase support for opposition parties [34] and lower the effective cost of participating, potentially leading to broader anti-regime action [35], as evidenced by the close correspondence between online protest activity and offline collective action [20].

A question that requires further exploration is whether the specific characteristics of the events we considered (such as their geographical location, the nature of the protests, the disruptive force of the actions taken) made them uniquely effective at activating a large periphery. Ultimately, core-periphery dynamics in online networks are the result of exogenous factors shaping the process; and yet, much in the same way as indexes of inequality like the Gini coefficient make it possible to compare countries that are in many respects incomparable, we believe that quantifying core-periphery dynamics can help us to compare episodes of large-scale social coordination, even if they arise in very different circumstances. Future work should consider alternative data sets and investigate whether the logic of our analysis can provide a solid basis for developing a taxonomy of social networks, which in turn would improve our theoretical understanding of the role that network structure plays in the growth of protest movements.

Prior research, for instance, demonstrates that cascading behavior in social networks depends greatly on the number of early adopters or first movers, but also (if more subtly) on how they are connected to one another and to the much larger community of potential participants [11]. This is the dimension that we try to capture with our core-periphery analyses. The distributions of influential and susceptible people in social networks, including the ways in which they cluster, is also important to explain propagation behavior [36]. We are still far from understanding how to best characterize these connections to reveal the association of network structure with cascading phenomena in collective action settings [22, 37]. Nevertheless, our findings indicate that we should give more theoretical prominence to the large periphery of people who generate most of the activity and help to increase the reach of messages. This is also important for more general studies of diffusion [38], which have focused on locating central or core individuals and have overlooked the significance of activating the critical periphery.

## Supporting Information

S1 Text Supplementary Materials. This document contains additional information about the estimation techniques, empirical analysis, and robustness tests.
(DOCX)
