# Supplementary material for: The Critical Periphery in the Growth of Social Protests
Source: PLoS One. 2015 Nov 30;10(11):e0143611. doi: 10.1371/journal.pone.0143611 (PMC4664236; doi:10.1371/journal.pone.0143611)
Supplement: S1 Text — This document contains additional information about the estimation techniques, empirical analysis, and robustness tests. (DOCX) [file pone.0143611.s001.docx]

Supplementary Materials

The Critical Periphery in the Growth of Social Protests

P. Barberá^a*^, N. Wang^b^, R. Bonneau^c^, J.T. Jost^d^, J. Nagler^a^, J. Tucker^a^, and S. González-Bailón^e*^

^a^ Department of Politics, New York University, New York, 10012, USA.

^b^ Mathematical Institute and Oxford Internet Institute, University of Oxford, Oxford, OX26GG, UK.

^c^ Center for Genomics and System Biology, New York University, New York, 10003, USA

^d^ Department of Psychology, New York University, New York, 10003 USA

^e^ Annenberg School for Communication, University of Pennsylvania, Philadelphia, 19104, USA.

*Correspondence to: [pablo.barbera@nyu.edu](mailto:pablo.barbera@nyu.edu); [sgonzalezbailon@asc.upenn.edu](mailto:sgonzalezbailon@asc.upenn.edu).

Contents

1. Bias in Twitter Data
2. Core-Periphery Analysis
3. Temporal Dynamics
4. References

1. Bias in Twitter Data.

Twitter’s publicly available APIs process a limited number of requests during a given time window and, as a result, do not return the full set of messages containing certain hashtags or keywords. Prior research has shown that the bias of the resulting sample depends on fluctuations in communication volume [i.e. samples are smaller when activity is higher; see (*1*)] and that the bias is more likely to affect the periphery of the network, that is, the representation of the less central users (*2*). Because of this bias, we are probably underestimating the importance of the periphery (to the extent that peripheral users are less likely to be sampled) and the number of retweets (or edges, including their weights), hence making our characterization of the critical periphery a conservative one. Another source of bias results from the fact that messages that are relevant to the protests do not always include hashtags, which we use as our search criteria to collect data. As a result, we are also probably underestimating overall activity volume. The effect of these biases can only be properly assessed with full access to the Twitter archive, currently behind a paywall.

2. Core-Periphery Analysis

A *k*-core is a maximal subgraph in which each vertex has at least degree *k (2, 3)*. As we describe in the main text of this article, the *k*-core decomposition is a recursive approach that progressively trims the least connected nodes in a network (i.e. those with lower degree) in order to identify its core. At the base of the decomposition procedure lie the most peripheral nodes; at the highest shells, those that are cohesively most central.

The output of this decomposition technique depends on the density and the degree distribution of networks. Figure S1 compares the *k*-cores in the same random network as in Figure 1 in the main text with those found in a lattice and a full graph with equal size. The degree distribution of these three networks vary significantly and so does, as a result, the classification of nodes in *k*-cores. In the lattice graph, all nodes are in the 2-core; in the full graph, all nodes are in the 19-core.

Empirical networks are obviously neither random, nor regular, nor fully connected, hence the importance of measuring their core-periphery structure: it helps uncover one important dimension of the interdependencies networks create as they emerge in real-world settings. Figure S2 compares the distribution of nodes in *k*-cores for the three networks we analyze with random networks that preserve the same size and density, and the same sequence for indegree and outdegree. Panels A, D, and G compare one random network with the observed network for the Gezi, Occupy, and Indignados datasets, respectively. As the plots show, the fraction of nodes classified in each *k*-shell is similar for most of the range, but the randomly generated networks underestimate the size and density of the core. In other words, they underestimate the cohesiveness and centrality of the minority as observed in empirical communication networks. Compared to a 1,000 randomly generated networks with the same constraints, the total number of *k*-cores varies depending on the random realization, but most of them are below the observed number (panels B, E and H); this is especially the case for the Gezi dataset where there are 158 cores but the random networks never contain more than 148. Finally, the highest *k*-core is significantly more populated in the observed networks than in the 1,000 random realizations, with the exception of the Indignados data (panels C, F and I).

Figure S3 shows the underlying adjacency matrices for the network of RTs displayed in figures 1 and 3 of the main text. The shade of each cell is proportional to the weight of the edges (or the aggregated number of RTs that members of one *k*-shell send to members of another). The matrices show that most of the RTs are sent from lower to higher *k*-cores (with higher levels of reciprocity at the periphery). This suggests that the vast majority of users are sourcing a significant amount of information from the minority of participants at the core of the networks. Figure S4 shows the adjacency matrices for the network of RTs in the Oscars data (panel A), and the minimum wage data Occupy data (panel B). The flow of information between core and peripheral users is sparser in both cases, especially for the Oscars network.

The relative size of core and periphery, and the communication dynamics that are created between the two, are important for a number of reasons. Network cores fulfill an integrative function: they provide multiple options for information flow and re-channeling. The periphery, on the other hand, is more loosely connected and has more fluctuations in activity levels. However, it also has fewer constraints to change, which has important implications for adaption to changing environments. These features have shown to be important in many of complex systems (*4*); in our context, they offer an x-ray of the self-organizing nature of political protest, and the ability for information to flow away from the core of committed participants.

In directed networks like ours, the decomposition can be based on indegree (i.e. incoming retweets), outdegree (i.e. outgoing retweets), or both to define the connectivity of a node. In our main analyses, we use both incoming and outgoing retweets to assign nodes to *k*-shells. As Fig. S5 shows, using incoming or outgoing ties results in less nested shells, but the distribution of core and peripheral participants is similar.

For the Gezi Park dataset, we also have information on the geolocation of some of the users, and the language in which Tweets were written. Location information was obtained by identifying tweets that contained the geographic coordinates from which they were sent. Language information is available with each individual tweet through the Twitter API and is based on a machine language detection algorithm designed by the company. As Fig. S6 shows, the periphery of the network contains more participants addressing an international audience, while the core has a higher percentage of users who are identified as being in the Taksim Gezi Park.

3. Temporal Dynamics

We distinguish core and peripheral participants using the structure of communication formed by retweets, which we further characterize using activity volume (or number of protest messages sent). There is yet another factor that is related to these core-periphery dynamics: time, or the length of participants’ commitment to protest communication. As Fig. S7 shows, participants that contribute for longer periods tend to be located at the center of the network –unsurprisingly, given than they accumulate more activity and have time to build denser connections. Most of the participants at the periphery, on the other hand, drop from the sample after one day of activity (which is not necessarily the same day, see Fig. S8). The percentage of participants that drop after one day is higher for the Occupy and Indignados networks, in line with their lower significance in terms of media impact compared to the Gezi Park case. These survival curves are based on the timing of all messages, not just retweets.

4. References

1. Morstatter F, Pfeffer J, Liu H, Carley KM (2013) Is the sample good enough? comparing data from twitter's streaming api with twitter's firehose. *Proceedings of the ICWSM* Conference, Boston, MA.

2. Seidman SB (1983) Network structure and minimum degree. *Social Networks* 5:269-287.

3. Alvarez-Hamelin JI, Dall'Asta L, Barrat A, Vespignani A (2005). Large scale networks fingerprinting and visualization using the k-core decomposition. *Advances in neural information processing systems*, pp. 41-50.

4. Csermely R, London A, Wu LY, Uzzi B (2013) Structure and dynamics of core/periphery networks. *Journal of Complex Networks* 1(2):93-123.


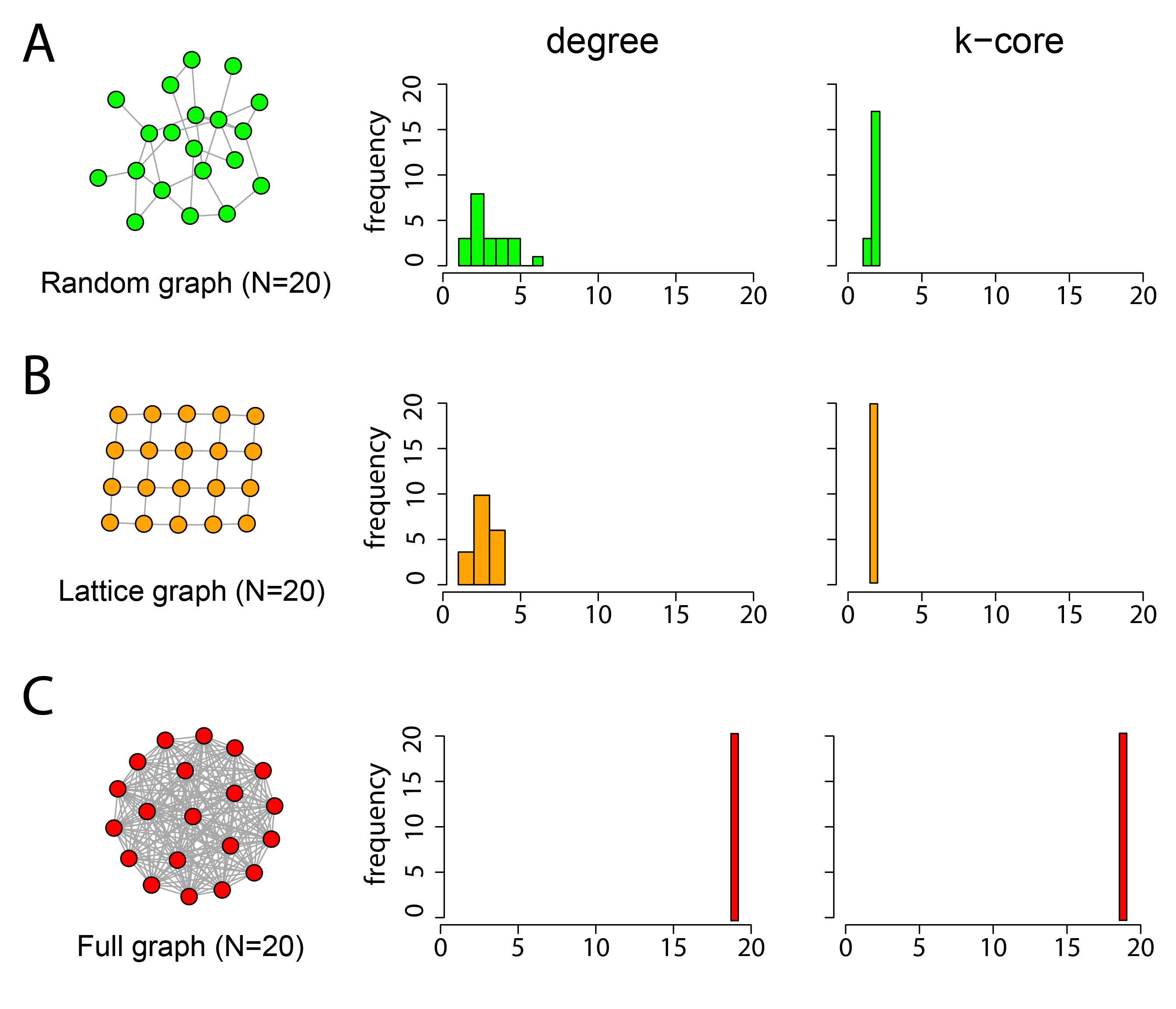


**Fig S1**. Example of *k*-core decomposition for three random networks of the same size but different density and degree distributions. All nodes in the lattice and full graph belong to a single *k*-core, but the cohesiveness of this core is (unsurprisingly) much higher in the fully connected network. According to this criterion, the random graph is a more hierarhical structure.

**
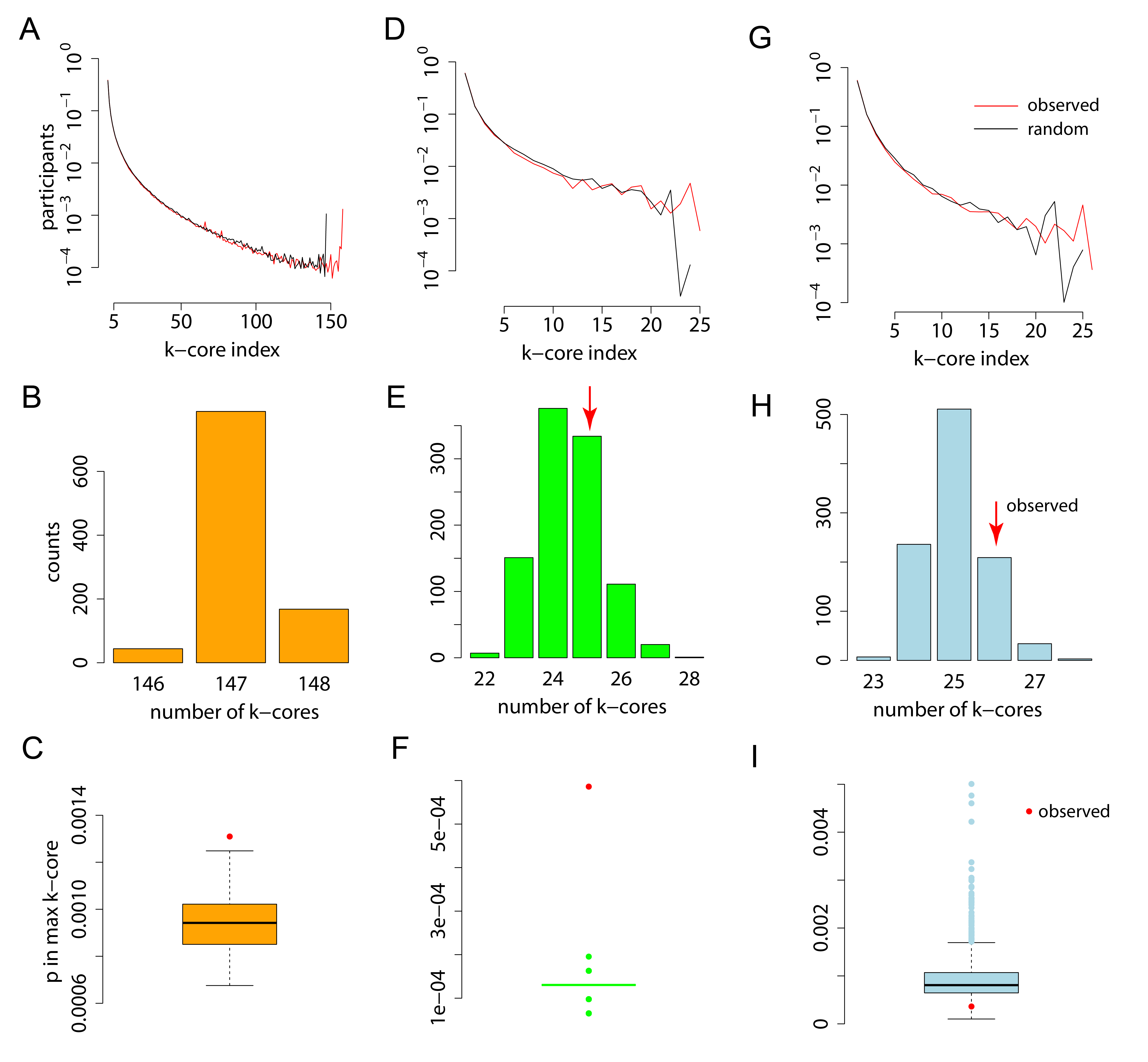
**

**Fig S2**. Distribution of nodes in *k*-cores for the three networks we analyze compared to random networks that preserve the same size, density, and sequence for indegree and outdegree (Gezi network in the first column; Occupy network in the second column; Indignados network in the third column). Panels A, D, and G compare the observed networks with one random realization. As the plots show, the fraction of nodes classified in each *k*-shell is similar for most of the range, but the randomly generated networks underestimate the size and density of the core. Compared to a 1,000 randomly generated networks, the total number of *k*-cores varies depending on the random realization, but most of them are below the observed number (panels B, E and H); this is especially the case for the Gezi dataset where there are 158 cores but the random networks never contain more than 148. The highest *k*-core is also significantly more populated in the observed networks than in the 1,000 random realizations, with the exception of the Indignados data (panels C, F and I).


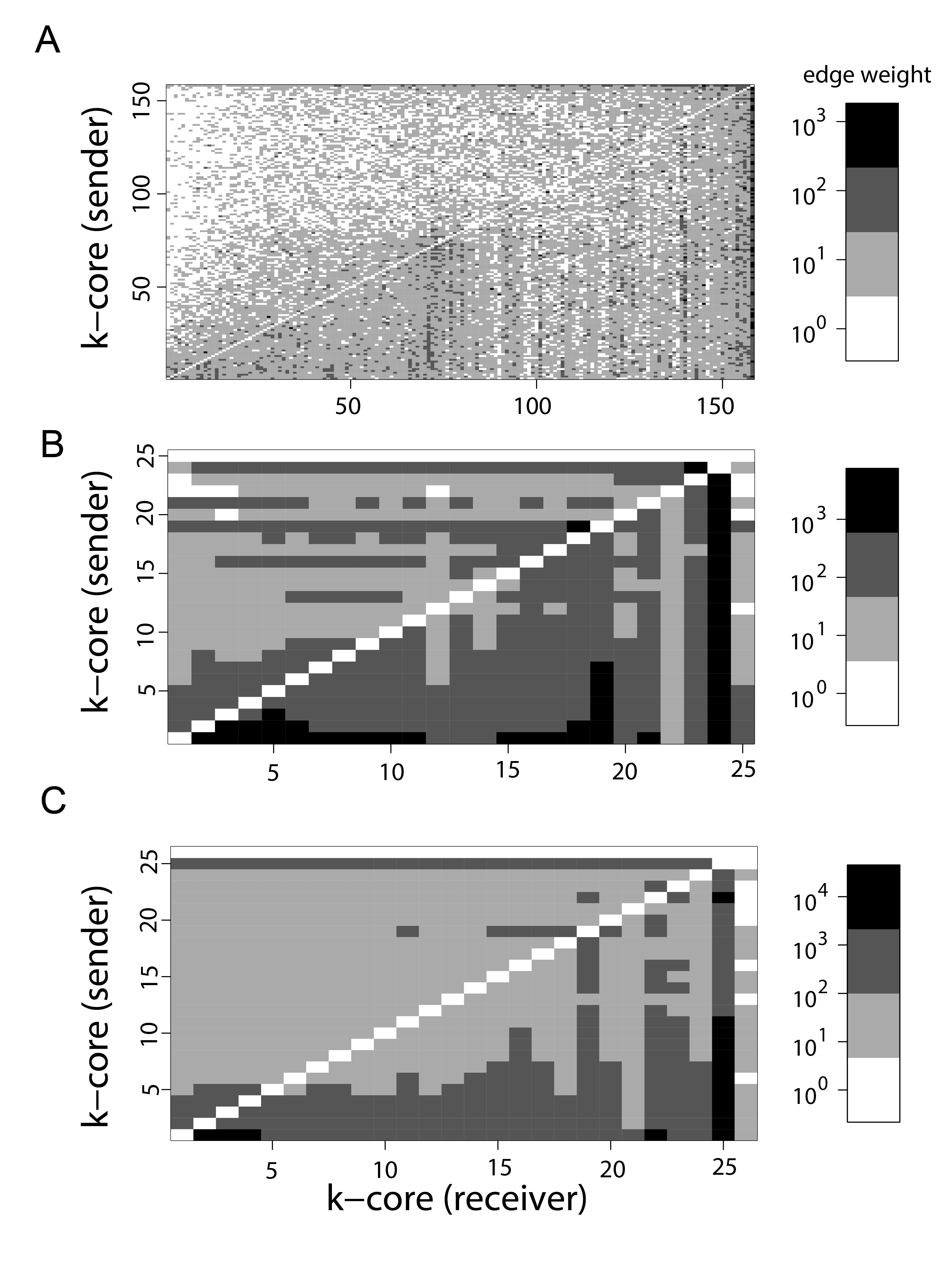


**Fig S3**. Adjacency matrices for the network of RTs in the Gezi data (panel A), the Occupy data (panel B), and the Indignados data (panel C). The shade of each cell is proportional to the weight of the edges (diagonals, the loops in the network, are empty to improve visualization). The matrices show that most of the RTs are sent from lower to higher *k*-cores, which means that the vast majority of users are sourcing information from the minority of participants at the core of the networks.


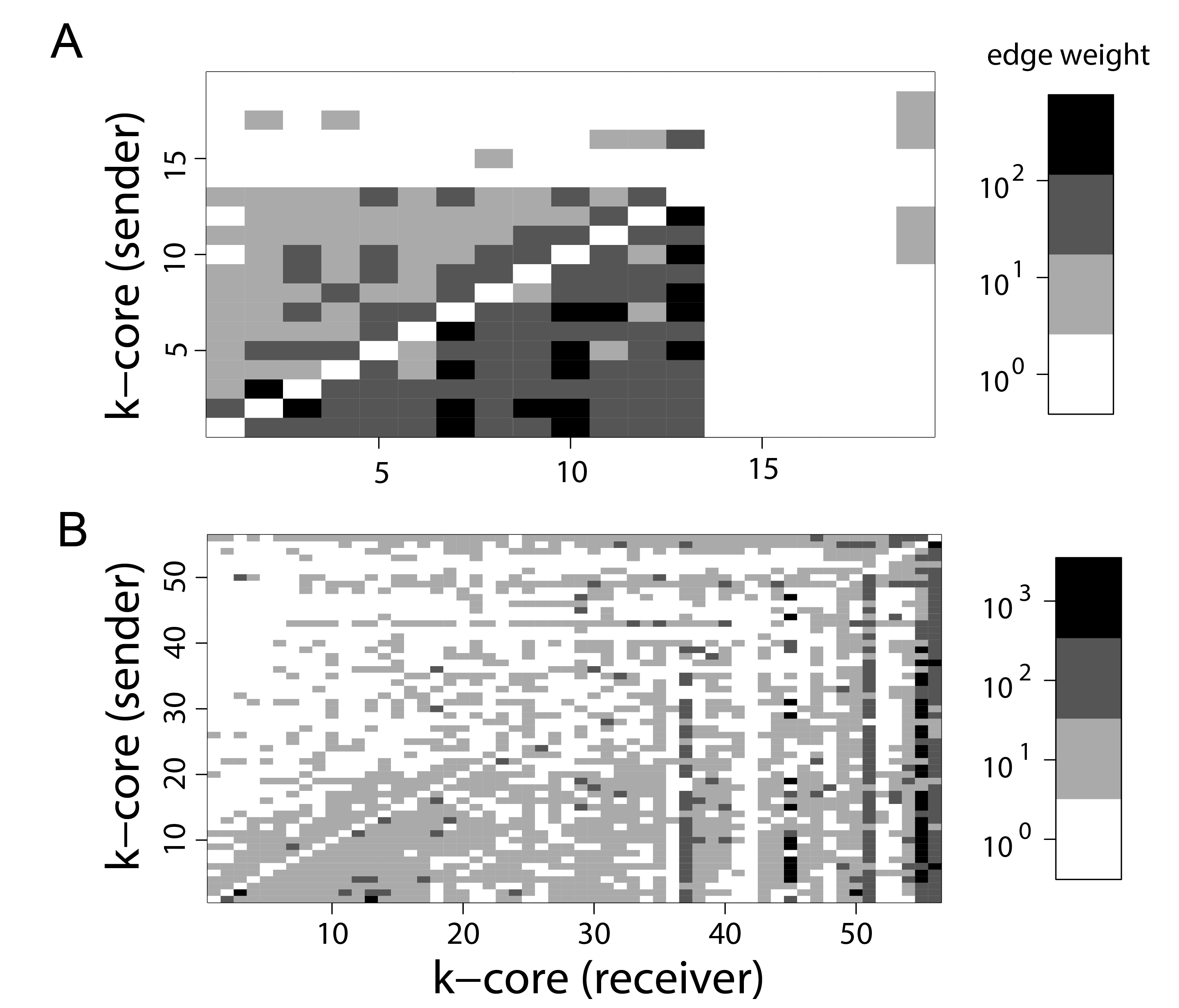


**Fig. S4.** Adjacency matrices for the network of RTs in the Oscars data (panel A), and the minimum wage data (panel B). The shade of each cell is proportional to the weight of the edges (diagonals, the loops in the network, are empty to improve visualization). The flow of information between core and peripheral users is lower in both cases compared to the protest networks, especially for the Oscars data.


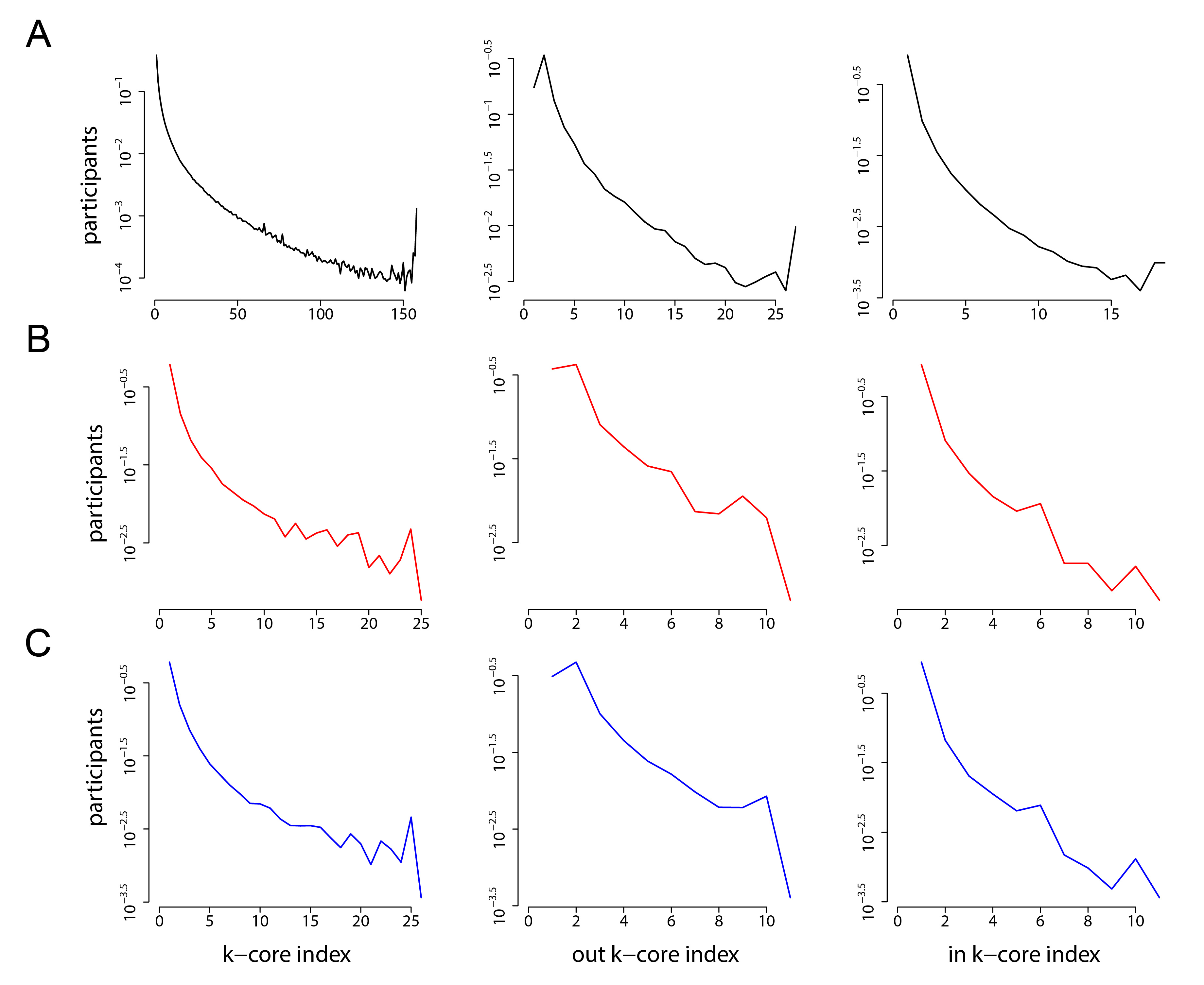


**Fig S5**. Fraction of participants in each *k*-shell for the Gezi network (row A), the Occupy network (row B), and the Indignados network (row C). Applying the decompostion to outdegree (outgoing retweets) and indegree (incoming retweets) results in a smaller number of nested layers but similar distribution of participants as core or peripheral.


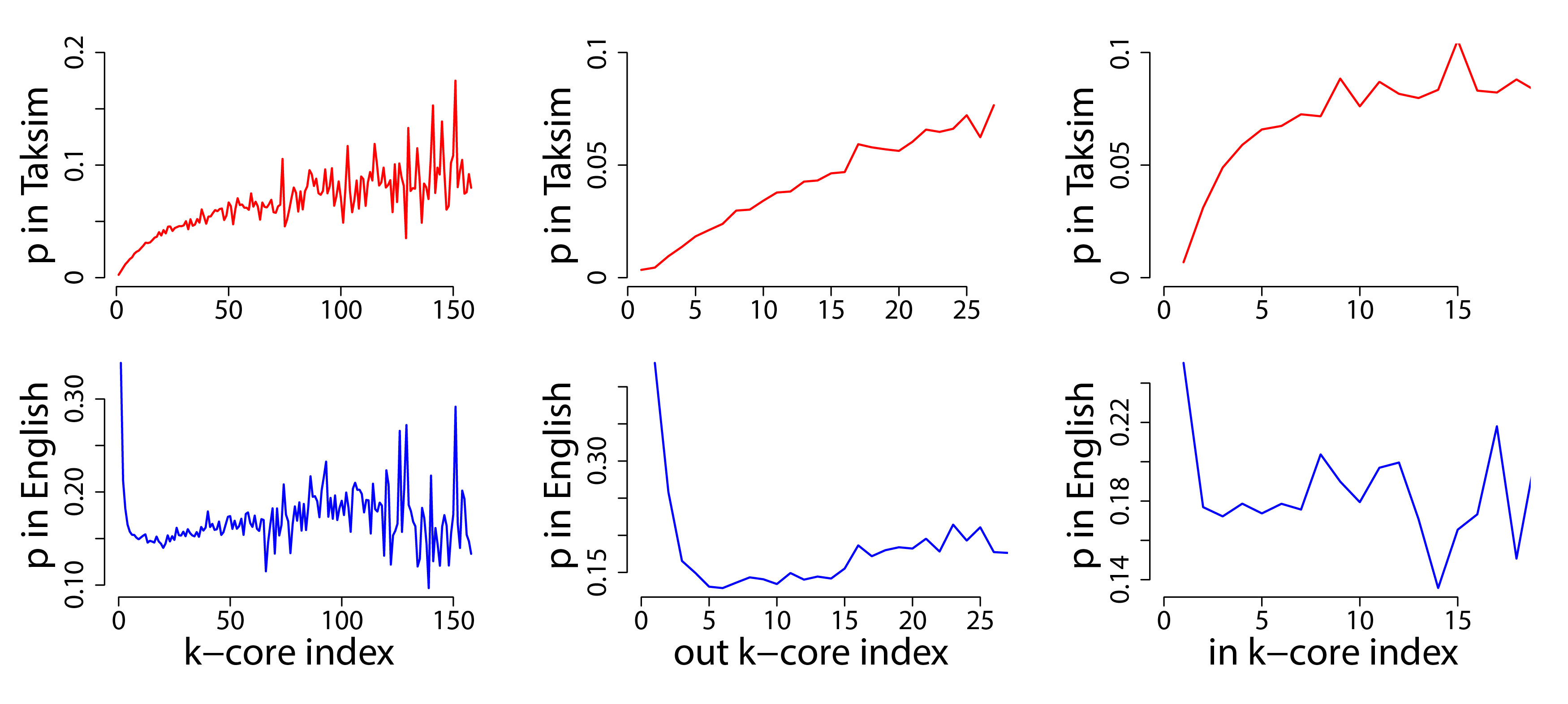


**Fig S6**. Proportion of participants in the Turkish protests that reported being in Taksim Gezi park (upper panels), and proportion of participants who wrote most of their Tweets in English (lower panels). The periphery of the network contains most of the participants that addressed an international audience, while the core has a higher percentage of users who reported being in the geographical epicenter of the protests.


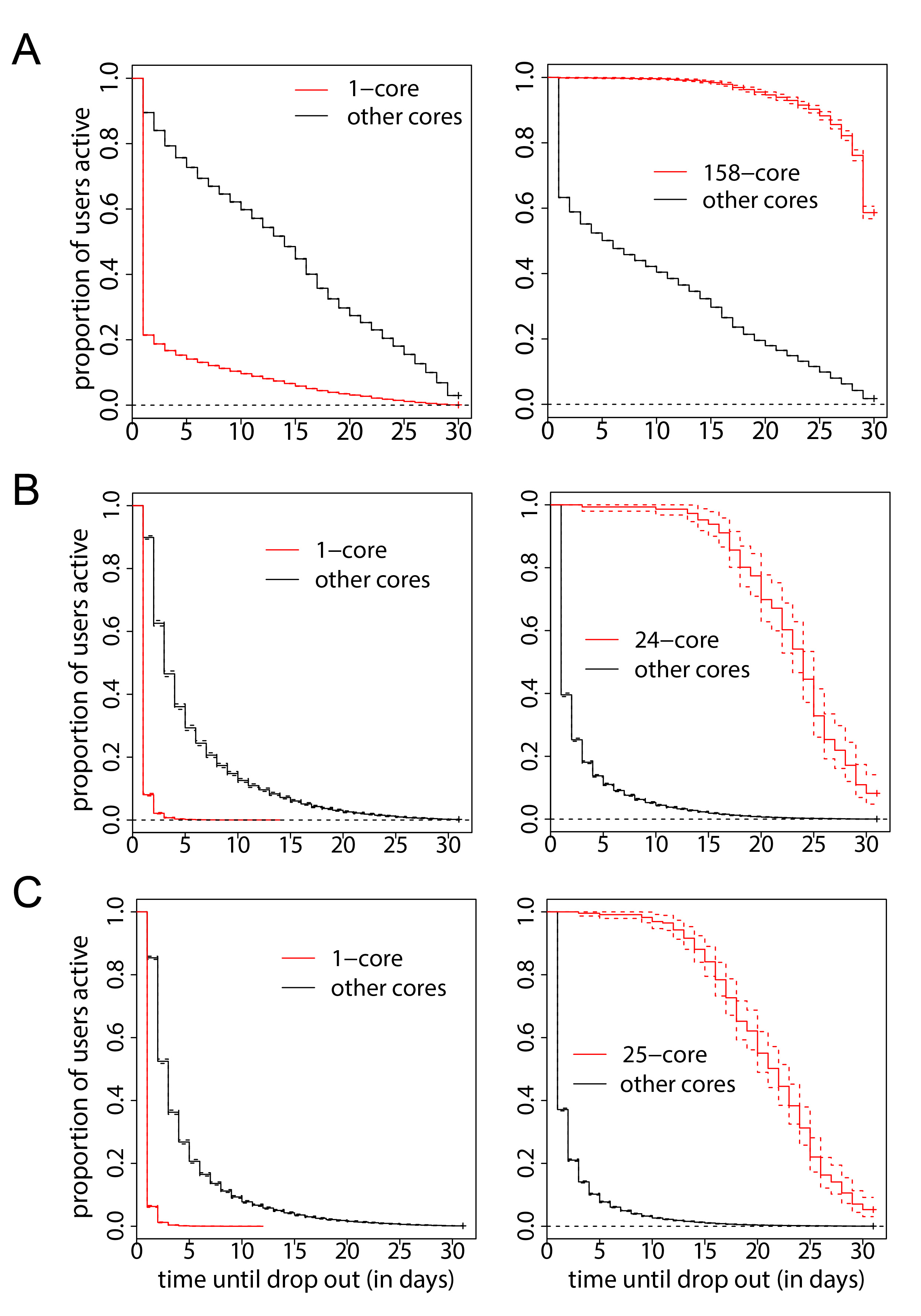


**Fig. S7**. Survival curves (and confidence intervals) for the Gezi network (row A), the Occupy network (row B), and the Indignados network (row C). Participants at the core were engaged in the exchange of information for longer periods.


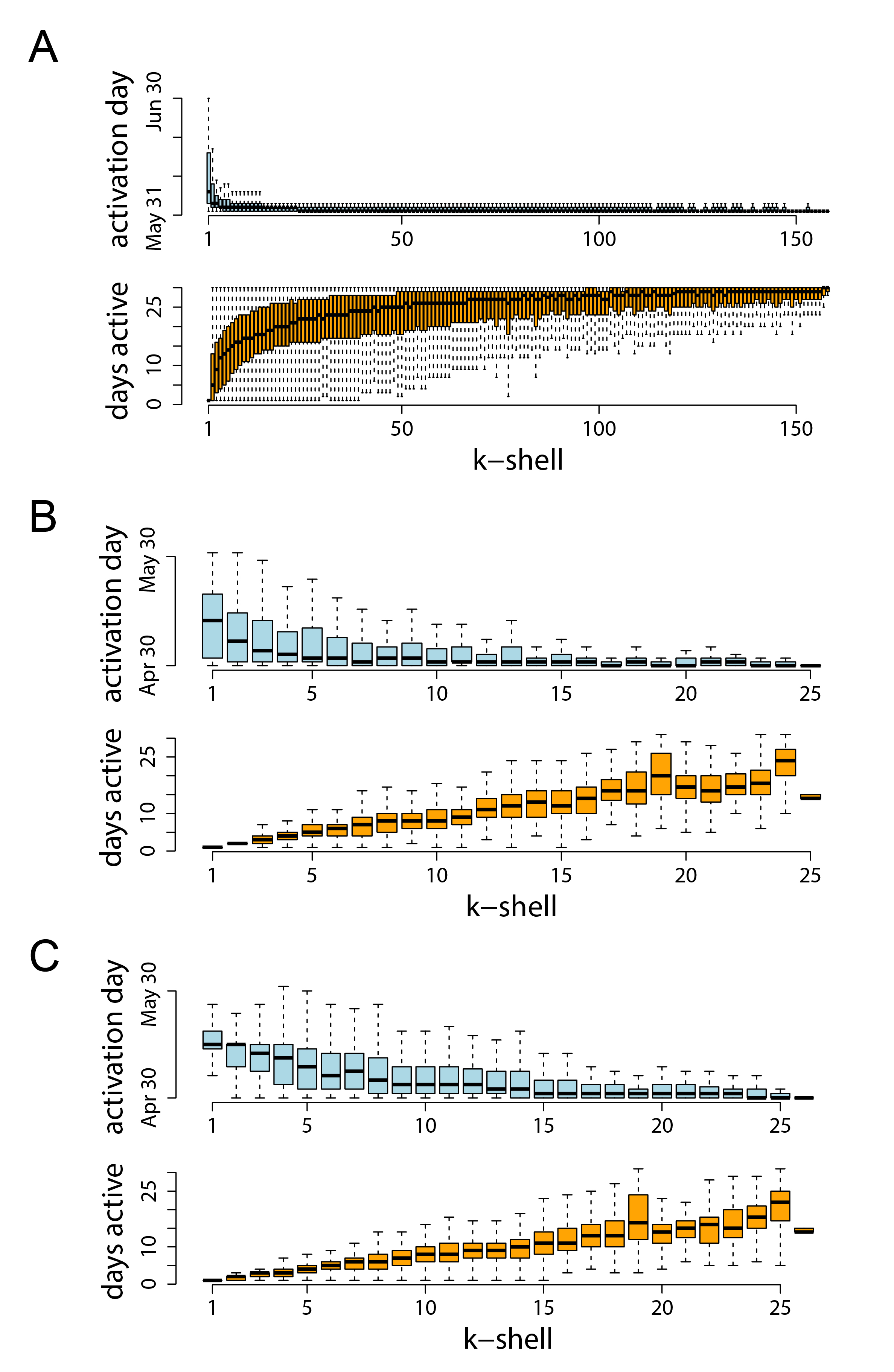


**Fig. S8**. Distribution of chronological activation time and days active for the Gezi network (panel A), the Occupy network (panel B), and the Indignados network (row C). Peripheral participants are engaged in the exchange of information for shorter periods, but became active at different points in time.
